# Mechanism of Cyclic Carbonate Synthesis from Epoxides and CO_2_**

**DOI:** 10.1002/anie.200805451

**Published:** 2009-04-06

**Authors:** Michael North, Riccardo Pasquale

**Affiliations:** * School of Chemistry, University of Newcastle upon Tyne, Bedson BuildingNewcastle upon Tyne, NE1 7RU (UK), Fax: (+44) 870-131-3783 E-mail: michael.north@ncl.ac.uk

**Keywords:** aluminum, carbon dioxide fixation, homogeneous catalysis, oxygen heterocycles, Schiff bases

**Scheme 1:**
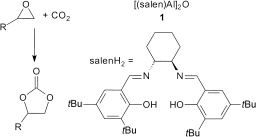
Synthesis of cyclic carbonates from epoxides and CO_2_.

There is currently much interest in the use of carbon dioxide as a chemical starting material, both to provide an alternative feedstock to fossil fuels and to help to mitigate global warming.[1] For the latter application, it is desirable that processes are developed which operate at atmospheric pressure and at or near room temperature. One reaction attracting significant attention in this respect is the 100% atom-economical synthesis of cyclic carbonates by the insertion of carbon dioxide into an epoxide (Scheme 1), though most current catalysts for this process require the use of high reaction temperatures and/or high pressures of carbon dioxide.[1],[2] We recently reported the development of bimetallic aluminum(salen) complex **1**, which when used in conjunction with tetrabutylammonium bromide constitutes the only catalyst system capable of catalyzing the insertion of carbon dioxide into terminal epoxides at 1 atm (760 mmHg) and at ambient temperature.[3] These extremely mild reaction conditions have allowed us to carry out the first mechanistic study of this important reaction, revealing a previously unanticipated role for the tetrabutylammonium bromide in the catalytic cycle.

The mechanistic studies were initiated with a detailed analysis of the reaction kinetics. Our previous work with catalyst **1** was carried out under solvent-free conditions, [3] and all attempts to carry out reactions in conventional solvents failed to produce any cyclic carbonate product. However, catalyst **1** is known to catalyze the synthesis of propylene carbonate from propylene oxide. Therefore, propylene carbonate, which is a liquid at room temperature, must be a suitable solvent for cyclic carbonate synthesis. Based on this, we developed standard conditions for the kinetic study in which styrene oxide served as the substrate and propylene carbonate as the solvent. The progress of these reactions could be conveniently monitored either by analysis of reaction samples by GC or by in situ FTIR.

The general form of rate equation is given by Equation (1). By working under conditions where carbon dioxide is present in large excess, and noting that the concentrations of catalysts **1** and tetrabutylammonium bromide will be effectively constant during the reaction, this can be simplified to Equation (2). In the event, the reactions followed first-order kinetics, [4] and by varying the concentrations of carbon dioxide, catalyst **1**, and tetrabutylammonium bromide, we could determine the rate equation as Equation (3)


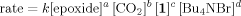
(1)


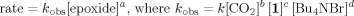
(2)


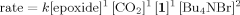
(3)

**Figure 1:**
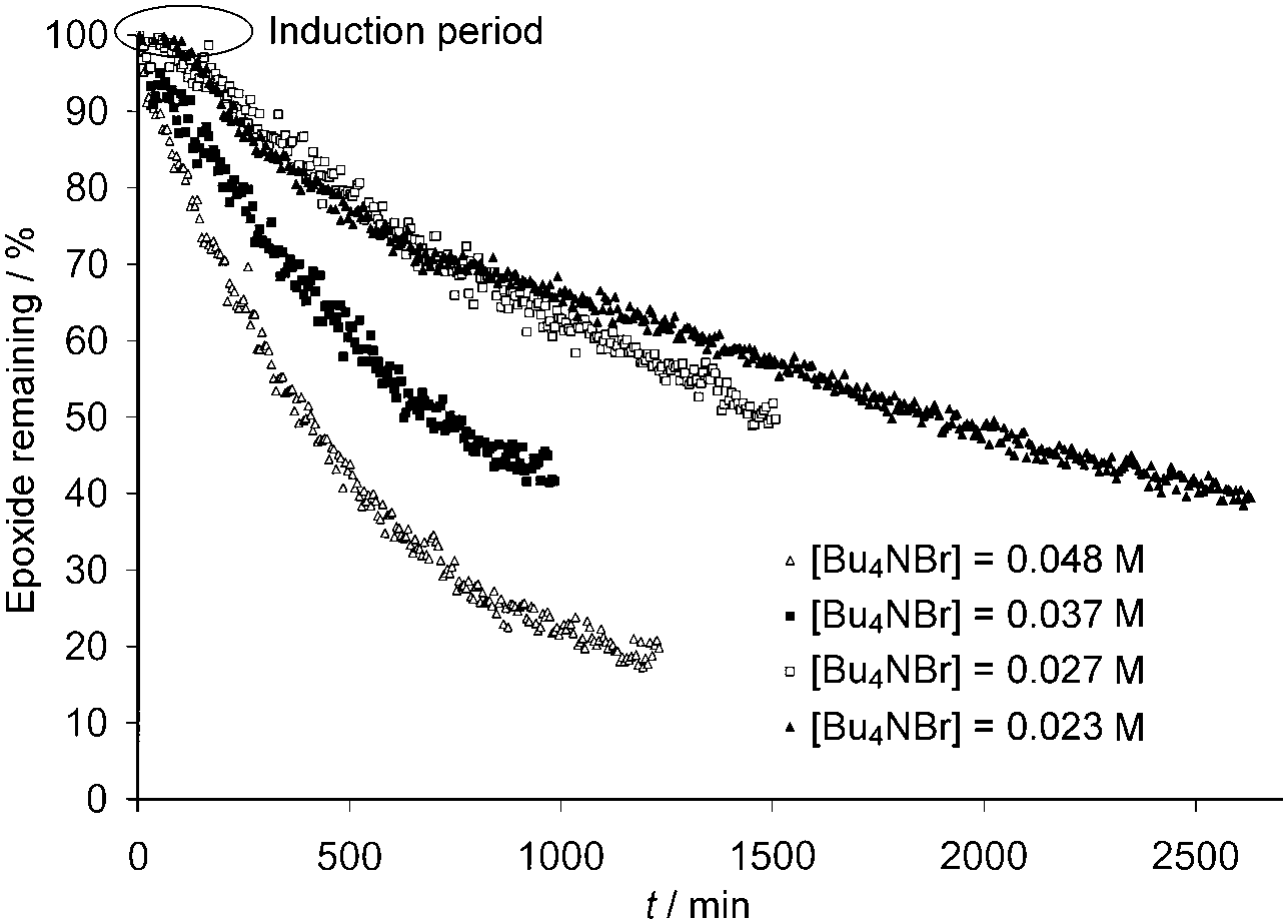
Decrease in styrene oxide concentration with time at various concentrations of tetrabutylammonium bromide at 26°C in propylene carbonate: [epoxide]_0_=1.6m, [**1**]=47 mm, excess CO_2_.

The first-order dependence on each reagent/catalyst concentration except tetrabutylammonium bromide was anticipated, but the second-order dependence of the rate on the tetrabutylammonium bromide cocatalyst concentration was unexpected and implies that two separate molecules of tetrabutylammonium bromide are involved in the catalytic cycle before the rate-determining step. It was also noted that reactions carried out at very low concentrations of tetrabutylammonium bromide had an induction period (Figure 1), though this was not apparent in reactions carried out at higher concentrations of tetrabutylammonium bromide.

**Scheme 2:**
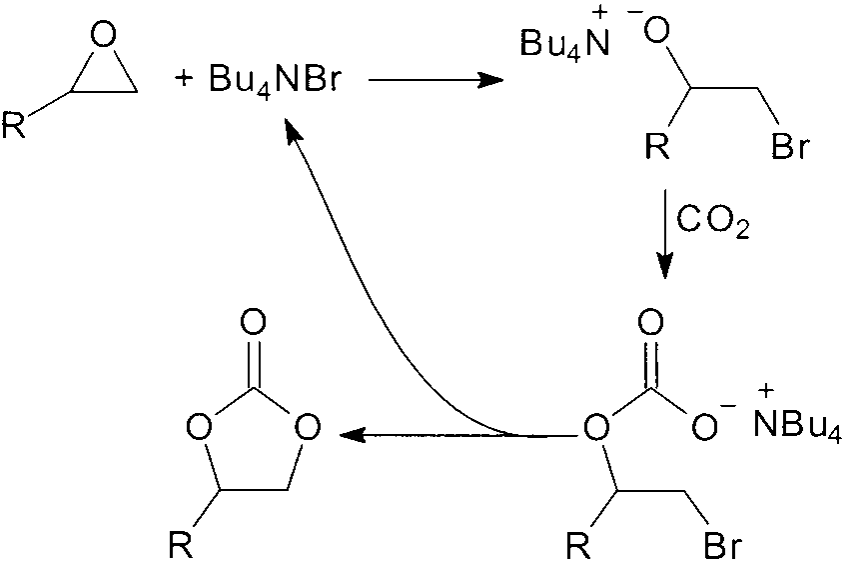
Known role of Bu_4_NBr in cyclic carbonate synthesis.

It is well established [5] that one role of tetrabutylammonium bromide and related species in cyclic carbonate synthesis is to open the epoxide ring to form a bromo-alkoxide (which can be stabilized by coordination to a metal catalyst such as **1**), which then reacts with carbon dioxide and cyclizes to give the cyclic carbonate with regeneration of the tetrabutylammonium bromide catalyst (Scheme 2). However, the kinetic data suggested that the tetrabutylammonium bromide was also involved in the mechanism in a second way.

Additional evidence on the role of tetrabutylammonium bromide came from reactions aimed at investigating the reusability of catalyst **1** and tetrabutylammonium bromide. These reactions were carried out under solvent-free conditions with propylene oxide as substrate and using 2.5 mol% of both salen complex **1** and tetrabutylammonium bromide as catalysts. After a reaction time of three hours under a carbon dioxide atmosphere, the propylene carbonate was distilled from the reaction flask and replaced with a new batch of propylene oxide and the process repeated. The purity of the propylene carbonate formed in these reactions was assayed by GC, and a second compound was sometimes found to be present. This was identified on the basis of its EI GC–MS as tributylamine. [4] The amount of tributylamine detected decreased as the catalysts were reused and eventually catalytic activity decreased. However, upon addition of more tetrabutylammonium bromide the catalytic activity was restored, and tributylamine was again detected in the propylene carbonate product. Control experiments showed that the tributylamine was not formed during the distillation process or by decomposition of tetrabutylammonium bromide within the GC–MS. [4]

Thus it appears that under the reaction conditions, tetrabutylammonium bromide decomposes to tributylamine by an S_N_2 [6] and/or E2 [7] mechanism. That tributylamine is a key component in the reaction and not just a decomposition product was confirmed by kinetics experiments carried out in the presence of catalyst **1**, tetrabutylammonium bromide, and tributylamine. [4] These experiments indicated that the rate of reaction depends on the concentration of all three species; and non-integer orders with respect to tetrabutylammonium bromide and tributylamine were observed, which is consistent with their interconversion under the reaction conditions.

**Scheme 3:**
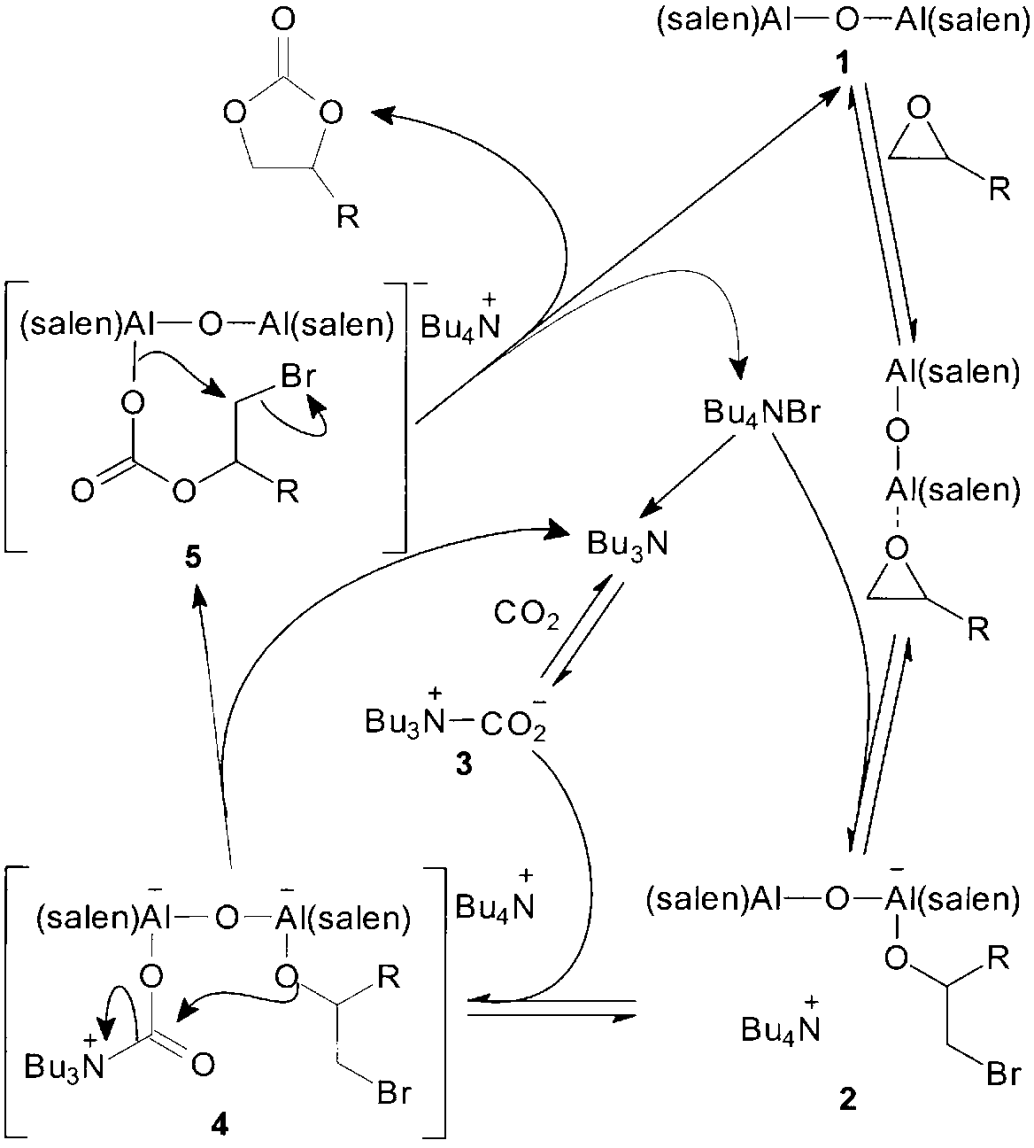
Catalytic cycle for cyclic carbonate synthesis.

Amines are well known to form carbamate salts with carbon dioxide; [1],[8] indeed this is the basis of many processes for the capture of carbon dioxide. These carbamate salts can be considered as activated forms of carbon dioxide and, compared to carbon dioxide itself, will also coordinate more readily to a metal complex such as **1**. Nucleophilic amines such as 4-dimethylaminopyridine (DMAP) have also been used to catalyze the formation of cyclic carbonates, [9] though formation of a trialkylamine in situ as part of the catalytic role of a tetraalkylammonium salt has not previously been considered. On the basis of this evidence, a catalytic cycle explaining how bimetallic complex **1** and tetrabutylammonium bromide combine to form a uniquely active system for cyclic carbonate can be proposed (Scheme 3).

In the catalytic cycle shown in Scheme 3, compound **1** first acts as a Lewis acid coordinating to the epoxide and activating it for ring-opening by bromide [10] provided by one tetrabutylammonium bromide molecule to generate species **2**. A second tetrabutylammonium bromide molecule is used to form tributylamine, which reacts reversibly with carbon dioxide to form carbamate salt **3**. The bimetallic nature of complex **2** then allows it to also coordinate carbamate **3** to give complex **4**. The ability to form complex **4**, in which both the epoxide and carbon dioxide have been activated and preorganized for intramolecular reaction, is a unique feature of catalysis by compound **1** and explains its exceptionally high level of catalytic activity. Mass spectrometry data confirm that catalyst **1** retains its bimetallic structure during reactions and after use in 16 consecutive reactions. [4] Displacement of the tributylammonium group from compound **4** generates the metal coordinated carbonate **5,** and subsequent ring-closure forms the cyclic carbonate and regenerates both catalyst **1** and tetrabutylammonium bromide. The second-order dependence on tetrabutylammonium bromide concentration (in the absence of added tributylamine) implies that formation of intermediate **4** is the rate-determining step of the catalytic cycle.

Given the widespread use of tetraalkylammonium salts (and related species such as tetraalkylphosphonium salts) as catalysts and cocatalysts in the formation of cyclic carbonates, it is likely that the roles of the tetrabutylammonium bromide outlined in Scheme 3 have general applicability to other catalyst systems. This is especially true of processes that operate at elevated temperatures as this is known to favor the conversion of tetraalkylammonium halides into trialkylamines. [6],[7]

In summary we have carried out a detailed kinetics analysis of cyclic carbonate synthesis catalyzed by the bimetallic aluminum(salen) complex **1**. As a result of the observed second-order dependence of the reaction rate on tetrabutylammonium bromide concentration and the identification of tributylamine in the reaction mixture, we have proposed a new catalytic cycle that fully explains the role of the tetraalkylammonium bromide cocatalyst.
