# Supplementary material for: Mechanism of Cyclic Carbonate Synthesis from Epoxides and CO2
Source: Angew Chem Int Ed Engl. 2009 Apr 6;48(16):2946–8. doi: 10.1002/anie.200805451 (PMC2898159; doi:10.1002/anie.200805451)
Supplement: Supplementary file 1 [file anie0048-2946-SD1.pdf]

Angewandte Chemie
Eine Zeitschrift der Gesellschaft Deutscher Chemiker

Supporting Information

© Wiley-VCH 2009

69451 Weinheim, Germany

SUPPORTING INFORMATION FOR:

Mechanism of cyclic carbonate synthesis from epoxides and CO_2

Michael North* and Riccardo Pasquale

School of Chemistry and University Research Centre in Catalysis and Intensified Processing, Bedson Building, Newcastle University, Newcastle upon Tyne, NE1 7RU, UK.

Contents

Details of chemicals and equipment 2

Experimental procedure for styrene carbonate synthesis monitored by in situ FTIR 2
Experimental procedure for styrene carbonate synthesis monitored by GC 3
Experimental procedure to allow the order with respect to carbon dioxide to be determined 3
Experimental procedure for the repeated use of catalyst **1** with propylene oxide 3
Control experiment on the use of tributylamine in the absence of Bu_4NBr 3

Kinetic analysis showing the reaction is first order in styrene oxide 5
Kinetic analysis showing the reaction is first order in catalyst **1** 6
Kinetic analysis showing the reaction is first order in carbon dioxide 7
Kinetic analysis showing the reaction is second order in tetrabutylammonium bromide 8

Kinetic analysis of the effect of varying the [Bu_4NBr] in the presence of Bu_3N 9
Kinetic analysis of the effect of varying the [Bu_3N] in the presence of Bu_4NBr 10

GCMS evidence for the presence of tributylamine 11
Sequential experiments showing the relationship between product formation and Bu_3N presence 13

Control experiments on the generation of tributylamine with detection by GCMS 14
Mass spectra of catalyst **1** before and after its use in 16 consecutive reactions 16

Details of chemicals and equipment

Commercially available chemicals (Alfa, Aldrich, Fluka) were used as received except for kinetics experiments. Glassware for kinetics experiments was dried in an oven overnight. Propylene carbonate and styrene oxide were freshly distilled prior to use. Catalyst **1** and tetrabutylammonium bromide were dried on a vacuum line at ca 50 ^{o}C for four hours prior to use. Chromatographic separations were performed using silica gel 60 (230-400 mesh, Davisil). In kinetics experiments with GC monitoring, aliquots were taken by SGE Type B 1 μL microsyringes. Distillations were carried out on a Büchi Kügelrohr GKR-50 apparatus. ^{1}H NMR spectra were recorded on Bruker Avance 300 and JEOL Lambda 500 spectrometers at 300 MHz and 500 MHz respectively. All spectra were recorded at room temperature. Electrospray mass-spectra were recorded on a Waters LCT Premier LCMS spectrometer (direct injection of the sample dissolved in MeOH).

In situ IR spectra were recorded using a Varian 800 FT-IR Scimitar series spectrometer fitted with a diamond tipped ATR (3 reflections) immersion probe. Spectra were scanned between 650 and 950 cm^{-1} with 80 scans being used to obtain each time point.

Gas chromatography was performed on a Varian CP-800 instrument with a TCD detector using a FactorFour (VF-1 ms) capillary column (15 m × 0.25 mm) with hydrogen as the carrier gas. The conditions used were: initial temperature 60 ^{o}C, hold at initial temperature for 2 minutes then ramp rate 15 ^{o}C/min to 270 ^{o}C; hold at final temperature for 5 minutes; T_R 3.52 minutes (styrene oxide), T_R 7.12 minutes (styrene carbonate). GCMS were recorded on a Varian CP-800-SATURN 2200 GC/MS ion-trap mass spectrometer using a FactorFour (VF-5 ms) capillary column (30 m × 0.25 mm) with helium as the carrier gas. The conditions used were: initial temperature 60 ^{o}C, hold at initial temperature for 3 minutes then ramp rate 15 ^{o}C/min to 270 ^{o}C; hold at final temperature for 5 minutes; T_R 7.33 minutes (styrene oxide), T_R 12.09 minutes (styrene carbonate). For the first 3.50 minutes, the eluent was routed away from the mass detector. Subsequently, the detector was operated in full EI scan mode.

All kinetics experiments were repeated at least twice and were mutually consistent. The GC response was calibrated with known mixtures of styrene oxide, styrene carbonate, propylene carbonate and tributylamine. The in situ FTIR was calibrated to monitor the disappearance of the styrene oxide peak at 873 cm^{-1}. A calibration table was constructed with four different concentrations of styrene oxide in propylene carbonate.

Styrene oxide concentration	Area of FTIR peak at 873 cm^{-1}
0	0
0.71 M	1.345
1.43 M	2.438
2.32 M	3.856

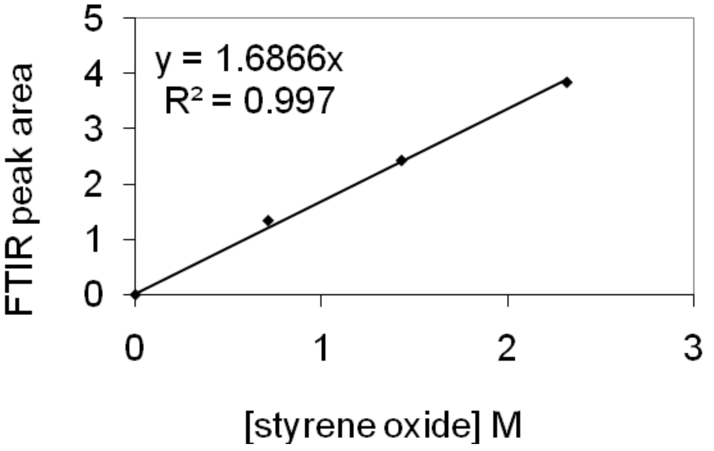

Experimental procedure for styrene carbonate synthesis monitored by in situ FTIR

Styrene oxide (0.83-5.48 mmol), complex **1** (0-0.09 mmol), tetrabutylammonium bromide (0-0.37 mmol) and tributylamine (0-0.04 mmol) were vigorously stirred in propylene carbonate (1.43 mL) until complete dissolution occurred. The mixture was placed in a 200 mL Schlenk flask fitted with the FTIR probe and equipped with a small magnetic stir-bar and placed in a thermostatted bath at 26 ^{o}C. An FTIR spectrum was recorded to calibrate the initial concentration of styrene oxide. The reaction flask was thoroughly flushed with CO_2 provided from a balloon inflated with several dry-ice pellets and was then mantained under a CO_2 atmosphere using the inflated balloon. A FTIR spectrum was recorded every 5.5 minutes. When the reaction had reached completion, the FTIR peak areas were converted into concentrations of styrene oxide using the calibration table and plotted against time to obtain the reaction profile.

Experimental procedure for styrene carbonate synthesis monitored by GC

Styrene oxide (0.83-2.74 mmol), complex **1** (0-0.04 mmol) and tetrabutylammonium bromide (0-0.02 mmol) were vigorously stirred in propylene carbonate (1.43 mL) until complete dissolution occurred. The mixture was placed in a 200 mL Schlenk flask equipped with a small magnetic stir-bar and placed in a thermostatted bath at 26 ^{o}C. A sample was withdrawn and analysed by GC to calibrate the initial concentration of styrene oxide. The reaction flask was thoroughly flushed with CO_2 provided from a balloon inflated with several dry-ice pellets and was then mantained under a CO_2 atmosphere using the inflated balloon. A sample was withdrawn for GC analysis every 30 minutes. When the reaction had reached completion, the GC peak areas were converted into concentrations of styrene oxide using the calibration table and plotted against time to obtain the reaction profile.

Experimental procedure to allow the order with respect to carbon dioxide to be determined

Styrene oxide (3.33 mmol, 0.400 g), complex **1** (0.09 mmol, 0.096 g) and tetrabutylammonium bromide (0.09 mmol, 0.028 g) were vigorously stirred in propylene carbonate (1.43 mL) until complete dissolution occurred. The mixture was placed in a 200 mL Schlenk flask fitted with the FTIR probe and equipped with a small magnetic stir-bar and placed in a thermostatted bath at 26 ^{o}C. An FTIR spectrum was recorded to calibrate the initial concentration of styrene oxide. Cylinders of CO_2 and N_2 were fitted with Cole-Parmer (0-250 mL/min) mass-flow control units and connected to a T-joint. The outlet from the T-joint was used to provide a stream of a known % of CO_2 in N_2 which passed through the reaction flask at atmospheric pressure and a FTIR spectrum was recorded every 5.5 minutes. When the reaction had reached completion, the FTIR peak areas were converted into concentrations of styrene oxide using the calibration table and plotted against time to obtain the reaction profile.

Experimental procedure for the repeated use of catalyst 1 with propylene oxide

Catalyst reusability experiments were carried out at 0 ^{o}C using propylene oxide as substrate. Complex **1** (2.5 mol%, 0.414 g) and tetrabutylammonium bromide (2.5 mol%, 0.115 g) were placed in a 25 mL round bottomed flask equipped with a magnetic stir bar and a SubaSeal. The mixture was placed in an ice-bath and left for 10 minutes until the system conditioned to 0 ^{o}C. At the same time, CO_2 was passed through the flask from a balloon inflated with several dry-ice pellets. Propylene oxide (14 mmol, 0.830 g) was then injected into the reaction and left to vigorously stir for 3 hours. The flask was then opened to air and left to warm to room temperature. The product was isolated directly from the reaction flask by micro-distillation under reduced pressure (0.2 Torr) using a Büchi Kugelrohr system at 140 ^{o}C and was obtained as a transparent liquid (yield 30-50 %) which was analysed by ^{1}HNMR and GC/MS. The catalyst left in the reaction flask was reused in the next cycle following the procedure reported above starting with cooling to 0 ^{o}C. After the completion of 16 cycles, a sample of the solid residue in the 25 mL flask was dissolved in methanol and analysed by high resolution electrospray mass spectrometry (positive ion mode) to show that the structure of catalyst **1** was unchanged.

Control experiment on the use of tributylamine in the absence of tetrabutylammonium bromide

Styrene oxide (0.40 g, 3.32 mmol), complex **1** (2.5 mol%, 0.10 g) and tributylamine (5 mol%, 0.20 g) were vigorously stirred in propylene carbonate (1.43 mL) until complete dissolution occurred. The mixture was placed in a 200 mL Schlenk flask equipped with a small magnetic stir-bar and placed in a thermostatted bath at 26 ^{o}C. A sample was withdrawn and analysed by GCMS to calibrate the initial concentration of styrene oxide. The reaction flask was thoroughly flushed with CO_2 provided from a balloon inflated with several dry-ice pellets and was then mantained under a CO_2 atmosphere using the inflated balloon. A sample was withdrawn for GCMS analysis after 21 hours which showed just 1.4% conversion of styrene oxide to styrene carbonate.

GC trace for a 1:1 mixture of styrene oxide and styrene carbonate

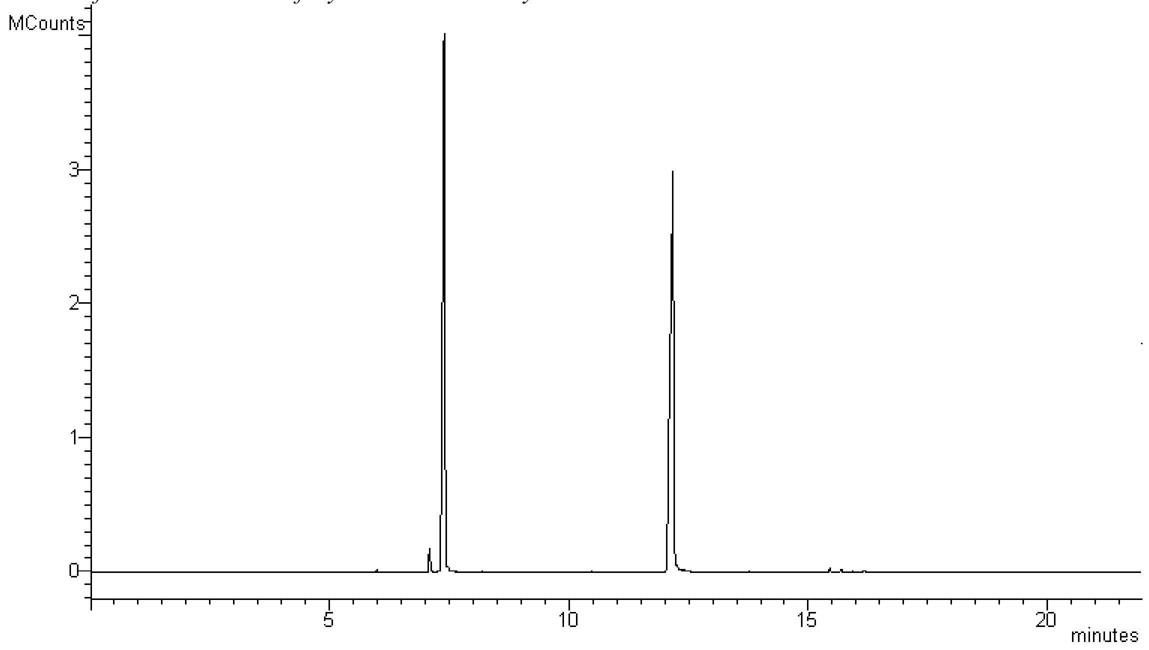

GC trace of control reaction mixture

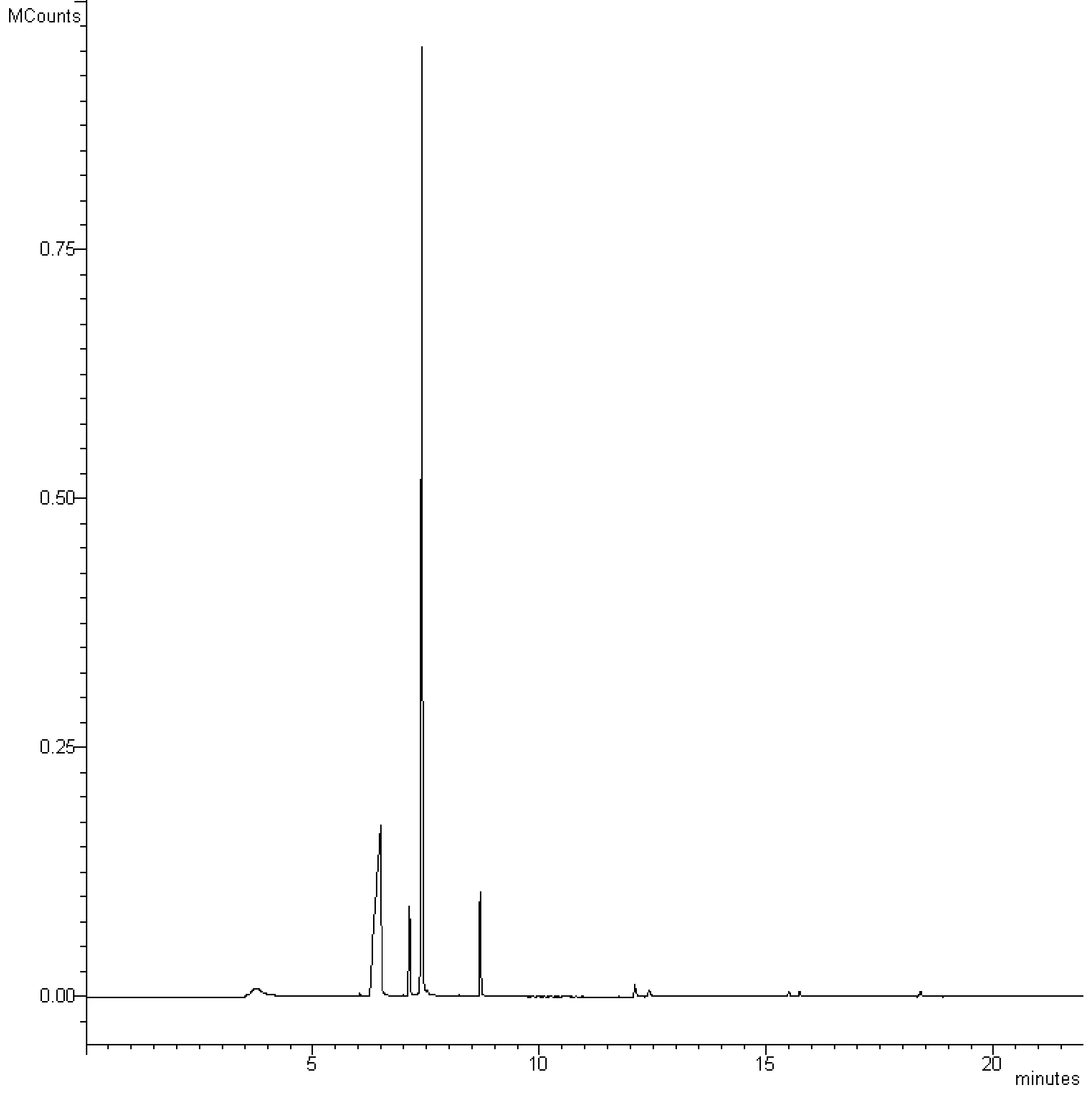

Kinetic analysis showing the reaction is first order in styrene oxide

First order kinetics plot at four different initial concentrations of styrene oxide.

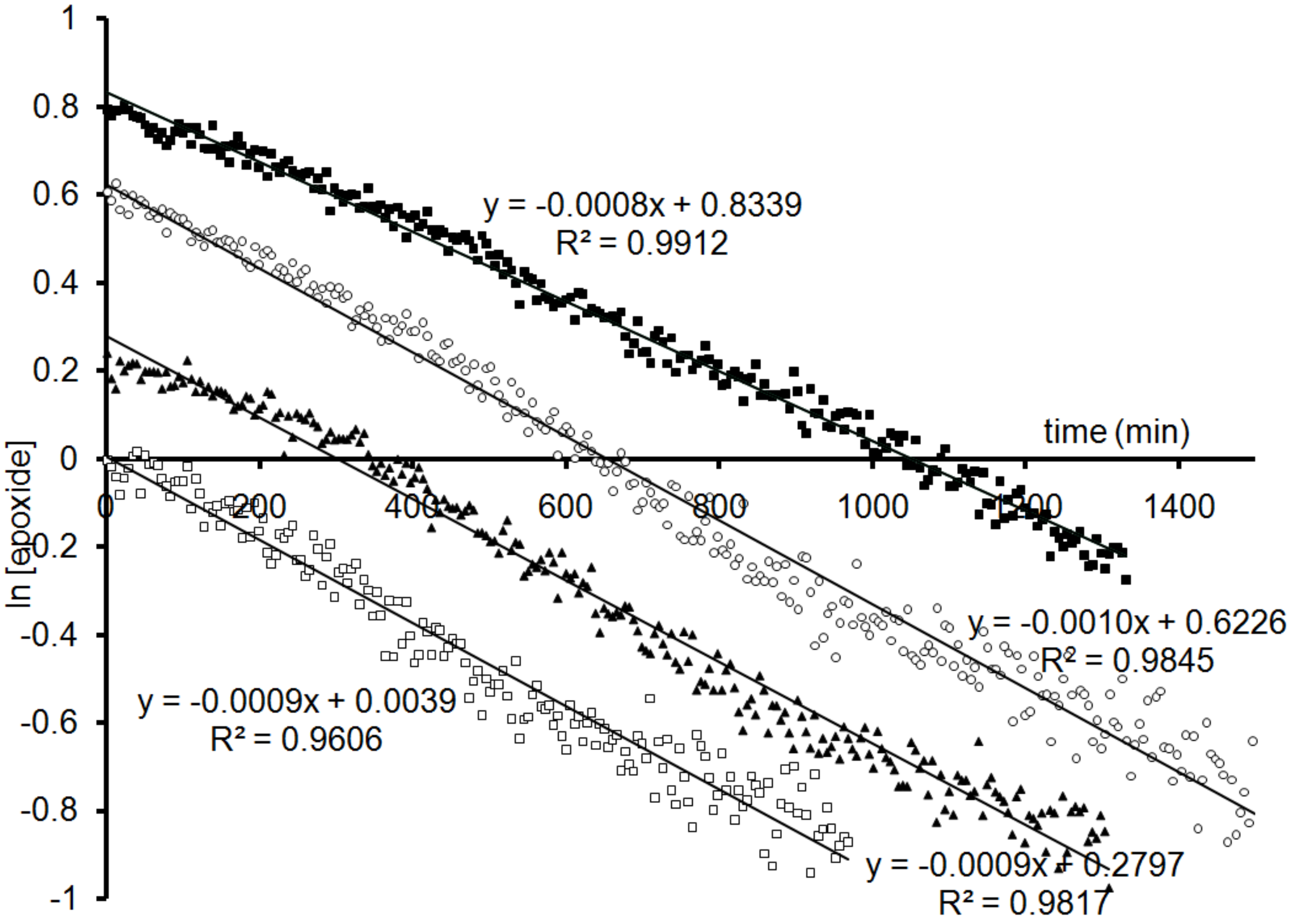

Filled squares [styrene oxide]$_0$ = 2.2 M, open circles [styrene oxide]$_0$ = 1.8 M, filled triangles [styrene oxide]$_0$ = 1.3 M, open squares [styrene oxide]$_0$ = 1.0 M. All reactions carried out at 26 ^oC with [**1**] = 46 mM, [Bu_4NBr] = 48 mM and in the presence of excess CO_2.

Kinetic analysis showing the reaction is first order in catalyst 1

*First order kinetics plot at four different concentrations of catalyst **1**.*

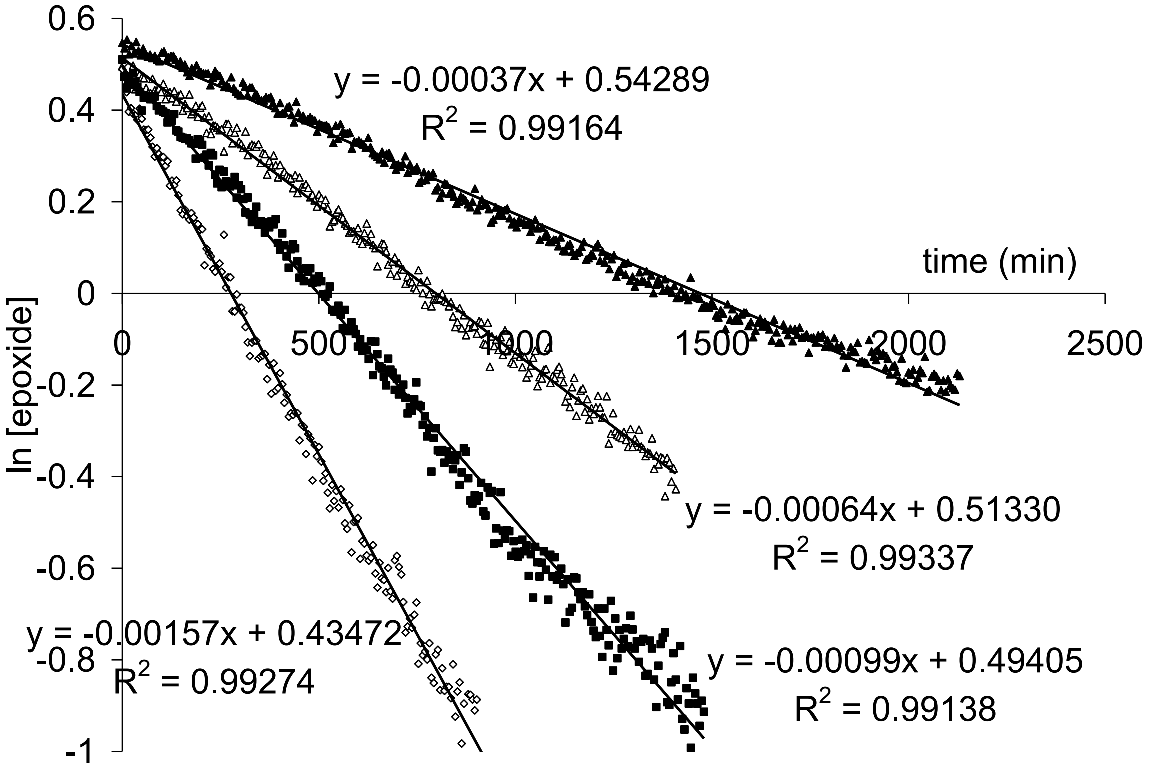

Filled triangles [**1**]= 13 mM, open triangles [**1**]= 19 mM, filled squares [**1**]= 34 mM, open diamonds [**1**]= 47 mM. All reactions carried out at 26 °C with $[\text{epoxide}]_0 = 1.6$ M, $[Bu_4NBr] = 48$ mM and in the presence of excess CO_2.

*Double logarithmic plot to determine the order with respect to catalyst **1**[a]*

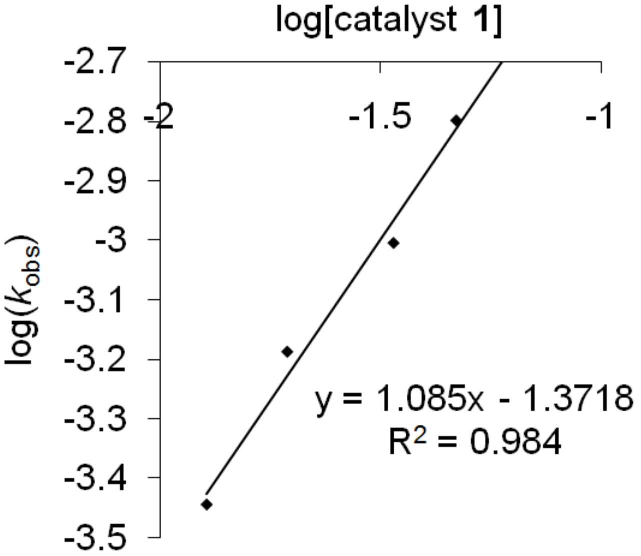

*Alternative non-logarithmic plot confirming that the reaction is first order in catalyst **1**[b]*

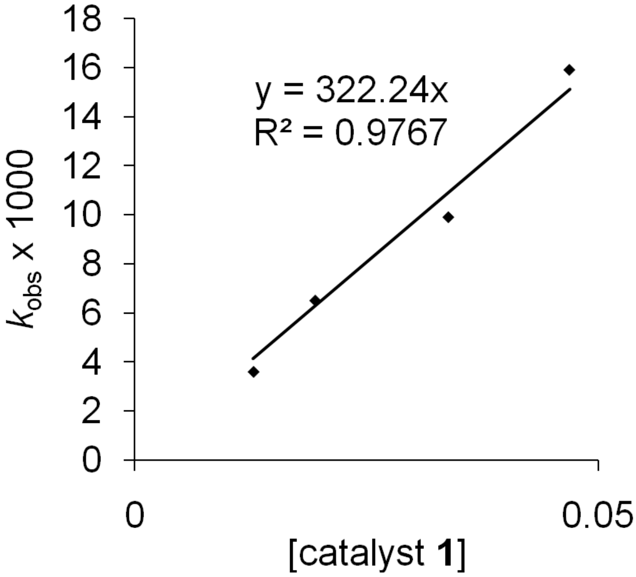

a) $k_{obs} = k[CO_2]^b[\mathbf{1}]^c[Bu_4NBr]^d$, so $\log(k_{obs}) = c \times \log[\mathbf{1}] + b \times \log[CO_2] + d \times \log[Bu_4NBr] + k$ and a plot of $\log(k_{obs})$ against $\log[\mathbf{1}]$ will have slope equal to b.
b) Assume reaction is first order in [**1**]. Then, $k_{obs} = k[\mathbf{1}]^1[CO_2]^b[Bu_4NBr]^d$, so a plot of k_{obs} against [**1**] should be a straight line with slope $k[Bu_4NBr]^d[CO_2]^b$ and passing through the origin

Kinetic analysis showing the reaction is first order in carbon dioxide

First order kinetics plot at five different concentrations of carbon dioxide.

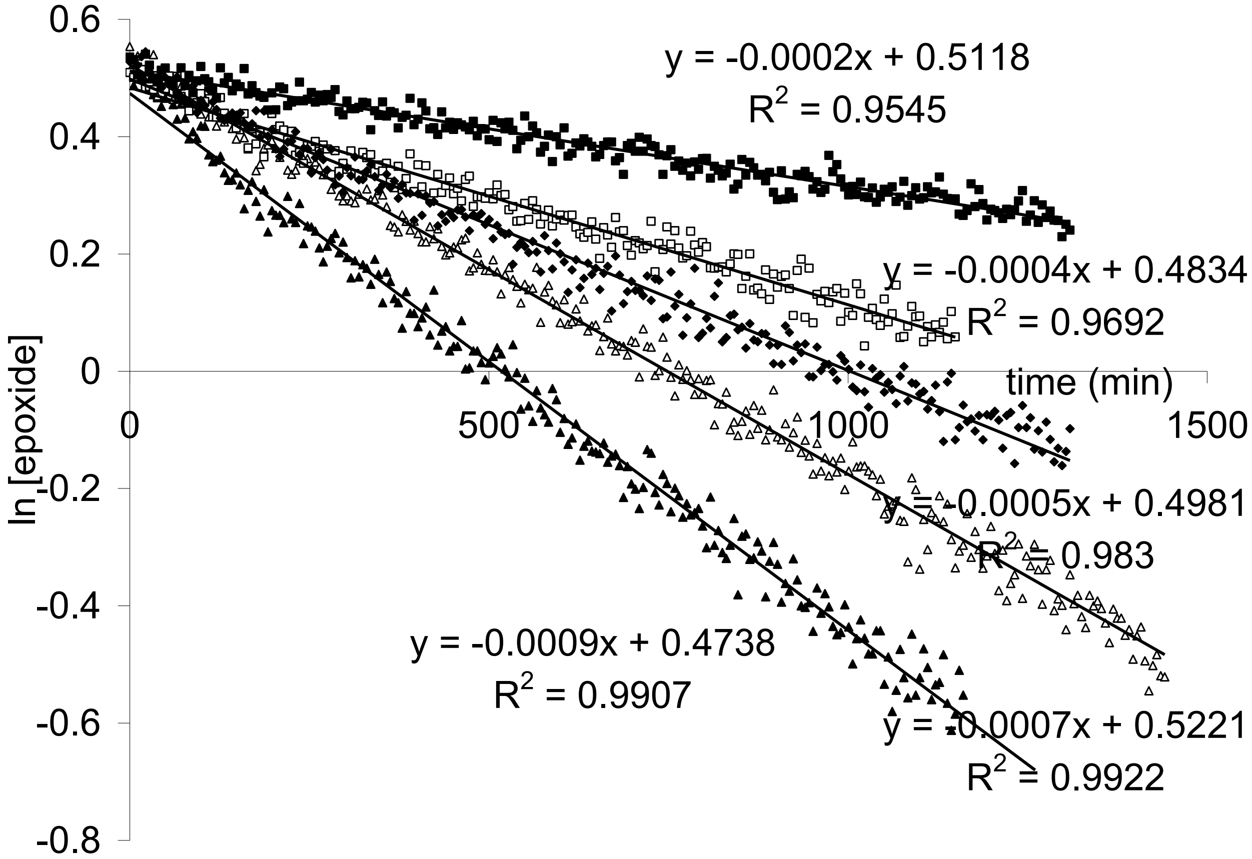

Filled squares 3.75% CO_2, open squares 7.1% CO_2, filled diamonds 11.0% CO_2, open triangles 14.3% CO_2, filled triangles 23.1% CO_2. All reactions carried out at 26 °C with $[\text{epoxide}]_0 = 1.7$ M, $[\mathbf{1}]_0 = 47$ mM and $[Bu_4NBr] = 48$ mM.

Double logarithmic plot to determine the order with respect to carbon dioxide[a]

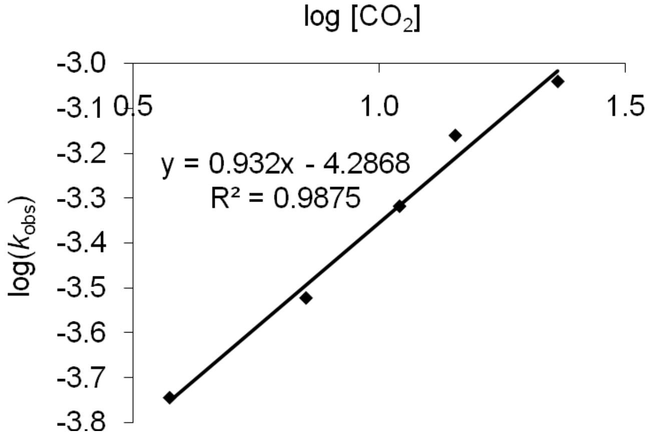

Alternative non-logarithmic plot confirming that the reaction is first order in carbon dioxide[b]

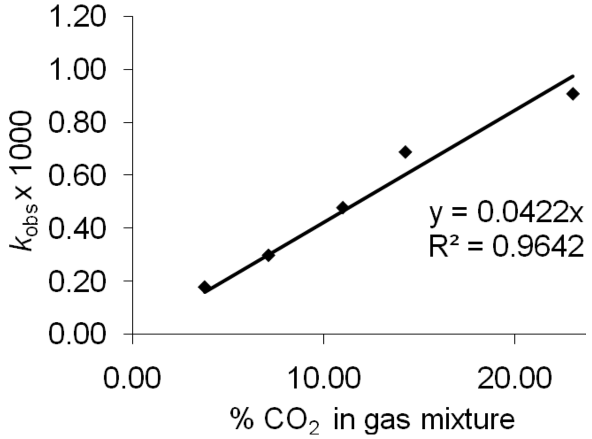

a) $k_{obs} = k[CO_2]^b[\mathbf{1}]^c[Bu_4NBr]^d$, so $\log(k_{obs}) = b \times \log[CO_2] + d \times \log[Bu_4NBr] + k + c \times \log[\mathbf{1}]$ and a plot of $\log(k_{obs})$ against $\log[CO_2]$ will have slope equal to b.

b) Assume reaction is first order in $[CO_2]$. Then, $k_{obs} = k[CO_2]^1[\mathbf{1}]^c[Bu_4NBr]^d$, so a plot of k_{obs} against $[CO_2]$ should be a straight line with slope $k[Bu_4NBr]^d[\mathbf{1}]^c$ and passing through the origin

Kinetic analysis showing the reaction is second order in tetrabutylammonium bromide

First order kinetics plot at four different concentrations of tetrabutylammonium bromide.

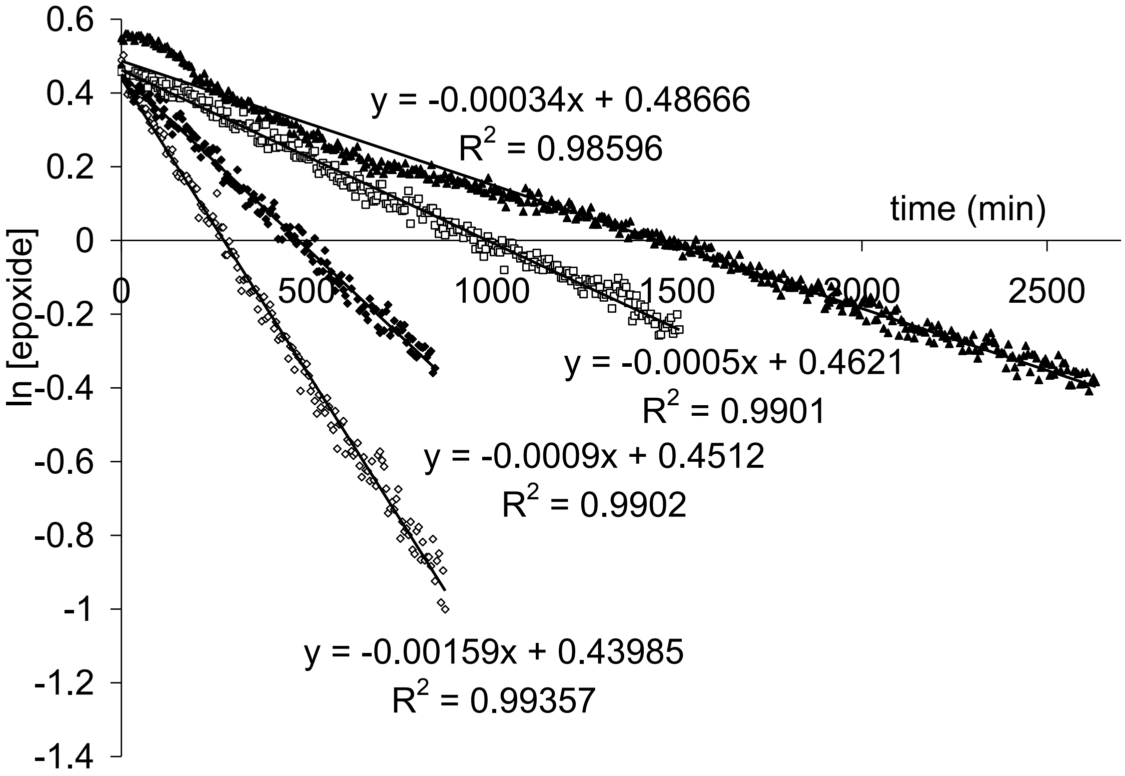

Filled triangles [Bu_4NBr]= 23 mM, open squares [Bu_4NBr]= 27 mM, filled diamonds [Bu_4NBr]= 37 mM, open diamonds [Bu_4NBr]= 48 mM. All reactions carried out at 26 °C with [epoxide]$_0$ = 1.6 M, [**1**] = 47 mM and in the presence of excess CO_2.

Double logarithmic plot to determine the order with respect to tetrabutylammonium bromide[a]

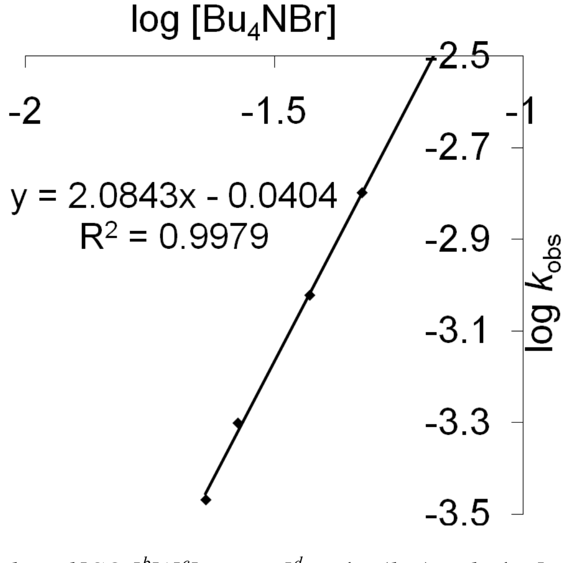

Alternative non-logarithmic plot confirming that the reaction is second order in [Bu_4NBr][b]

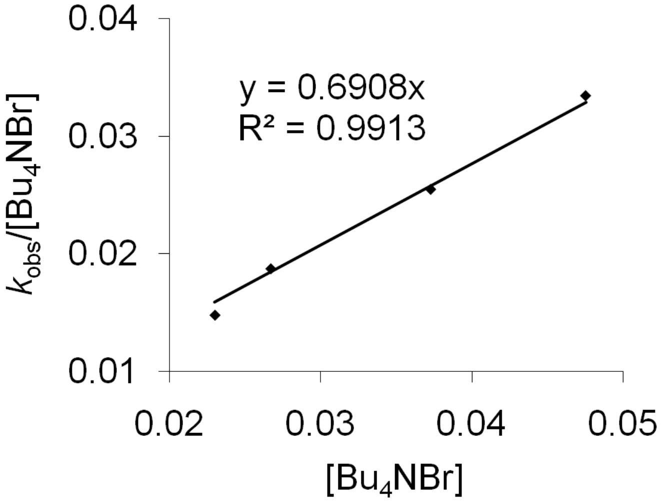

a) $k_{obs}= k[CO_2]^b[1]^c[Bu_4NBr]^d$, so $\log(k_{obs}) = d \times \log[Bu_4NBr] + k + b \times \log[CO_2] + c \times \log[\mathbf{1}]$ and a plot of $\log(k_{obs})$ against $\log[Bu_4NBr]$ will have slope equal to d.
b) Assume reaction is second order in [Bu_4NBr]. Then, $k_{obs}= k[CO_2]^b[\mathbf{1}]^c[Bu_4NBr]^2$ and $k_{obs}/[Bu_4NBr] = k[CO_2]^b[\mathbf{1}]^c[Bu_4NBr]$. So a plot of $k_{obs}/[Bu_4NBr]$ against [Bu_4NBr] should be a straight line with slope $k[CO_2]^b[\mathbf{1}]^c$ and passing through the origin

Kinetic analysis of the effect of varying the [Bu_4NBr] in the presence of Bu_3N

First order kinetics plot at four different concentrations of tetrabutylammonium bromide.

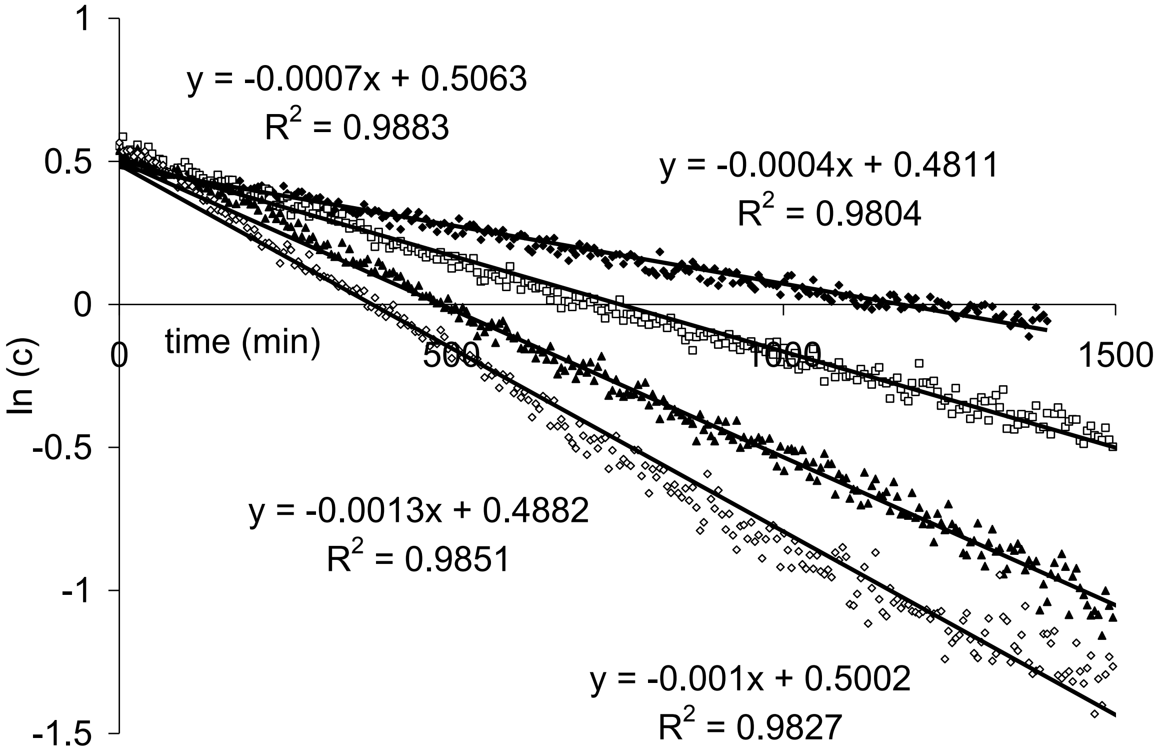

Filled diamonds [Bu_4NBr]= 22 mM, open squares [Bu_4NBr]= 46 mM, filled diamonds [Bu_4NBr]= 92 mM, open diamonds [Bu_4NBr]= 140 mM. All reactions carried out at 26 °C with [epoxide]$_0$ = 1.7 M, [**1**] = 45 mM, [Bu_3N]= 23 mM and in the presence of excess CO_2.

Double logarithmic plot to determine the order with respect to tetrabutylammonium bromide

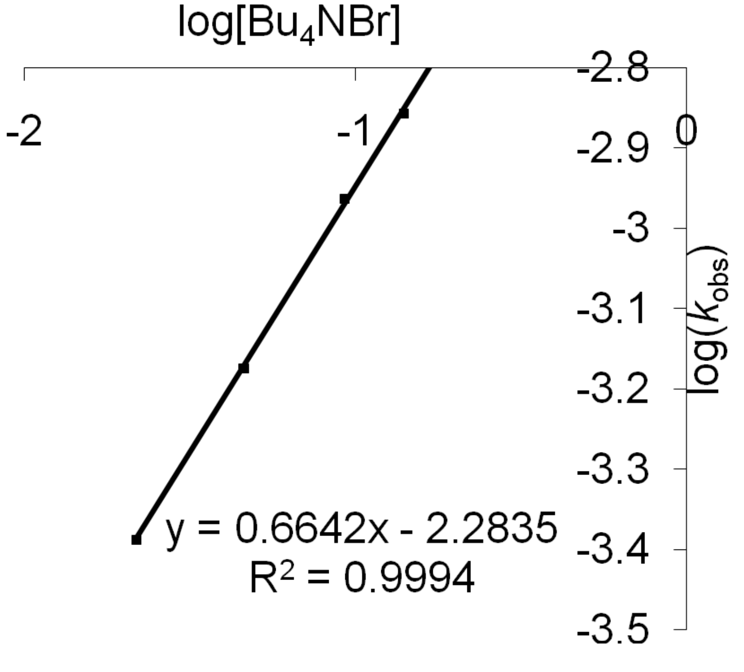

$k_{obs}= k[CO_2]^b[\mathbf{1}]^c[Bu_4NBr]^d[Bu_3N]^e$
so $\log(k_{obs}) = d \times \log[Bu_4NBr] + k + b \times \log[CO_2] + c \times \log[\mathbf{1}] + e \times \log[Bu_3N]$ and a plot of $\log(k_{obs})$ against $\log[Bu_4NBr]$ will have slope equal to *d*.

Kinetic analysis of the effect of varying the [Bu_3N] in the presence of Bu_4NBr

First order kinetics plot at three different concentrations of tributylamine.

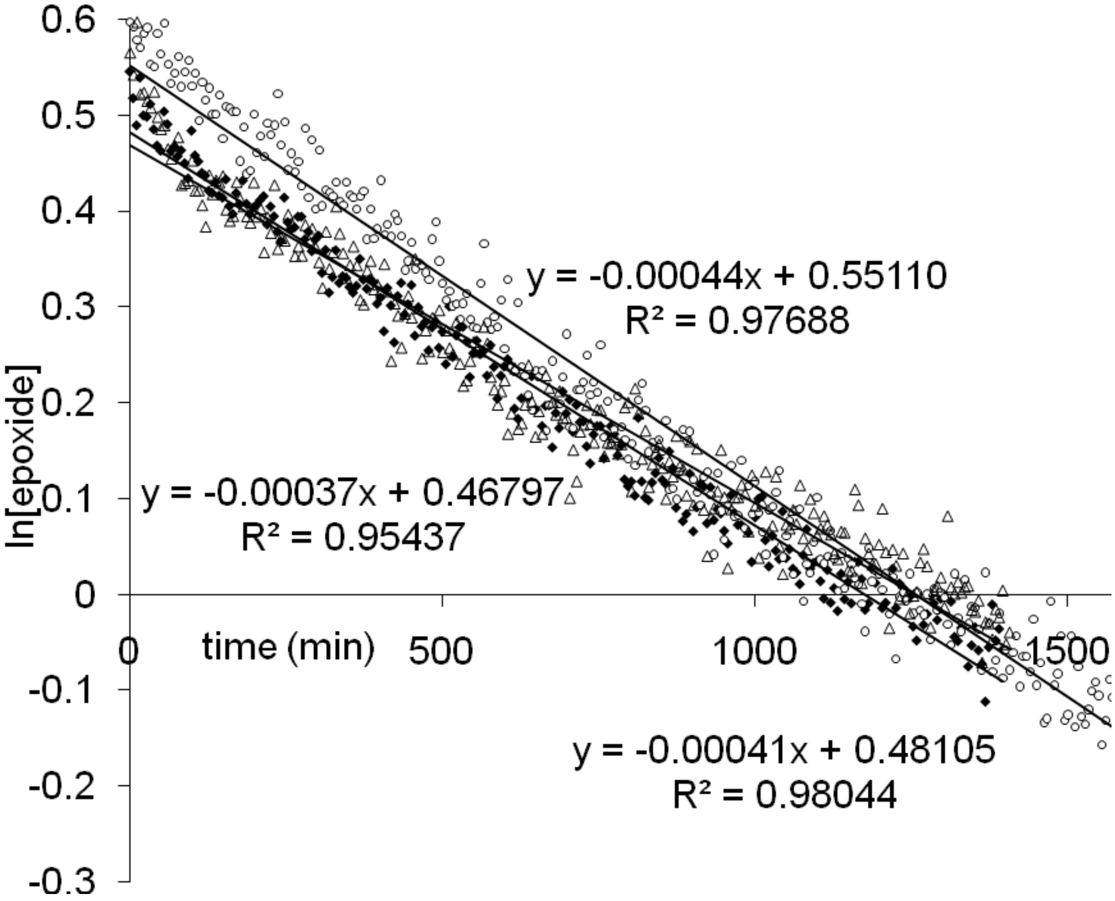

Open circles [Bu_3N]= 0.0447 M, filled squares [Bu_3N]= 0.0229 M, open triangles [Bu_3N]= 0.0119 M. All reactions carried out at 26 ^{o}C with [epoxide]$_0$ = 1.8 M, [**1**] = 46 mM, [Bu_4NBr]= 22 mM and in the presence of excess CO_2.

Double logarithmic plot to determine the order with respect to tributylamine

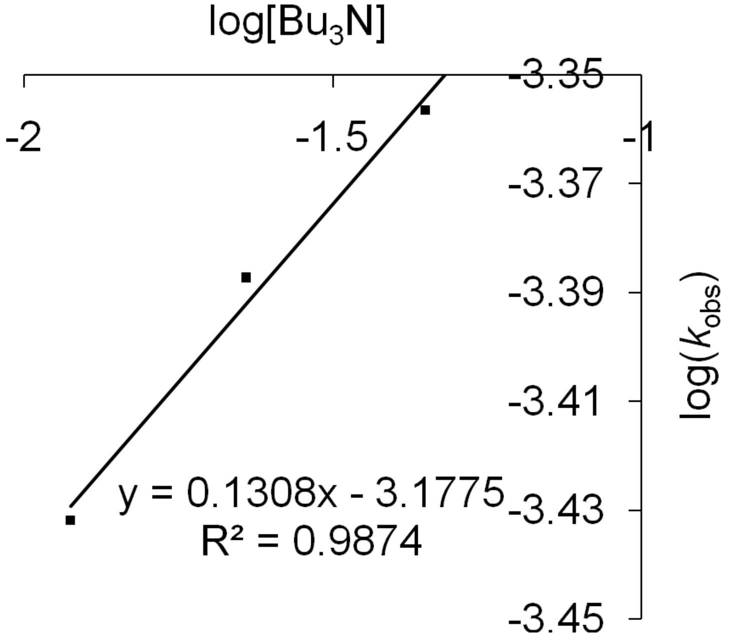

k_{obs}= $k[CO_2]^b[\mathbf{1}]^c[Bu_4NBr]^d[Bu_3N]^e$
so $\log(k_{obs}) = e \times \log[Bu_3N] + d \times \log[Bu_4NBr] + k + b \times \log[CO_2] + c \times \log[\mathbf{1}]$ and a plot of $\log(k_{obs})$ against $\log[Bu_3N]$ will have slope equal to e.

GCMS evidence for the presence of tributylamine

GC trace for authentic tributylamine

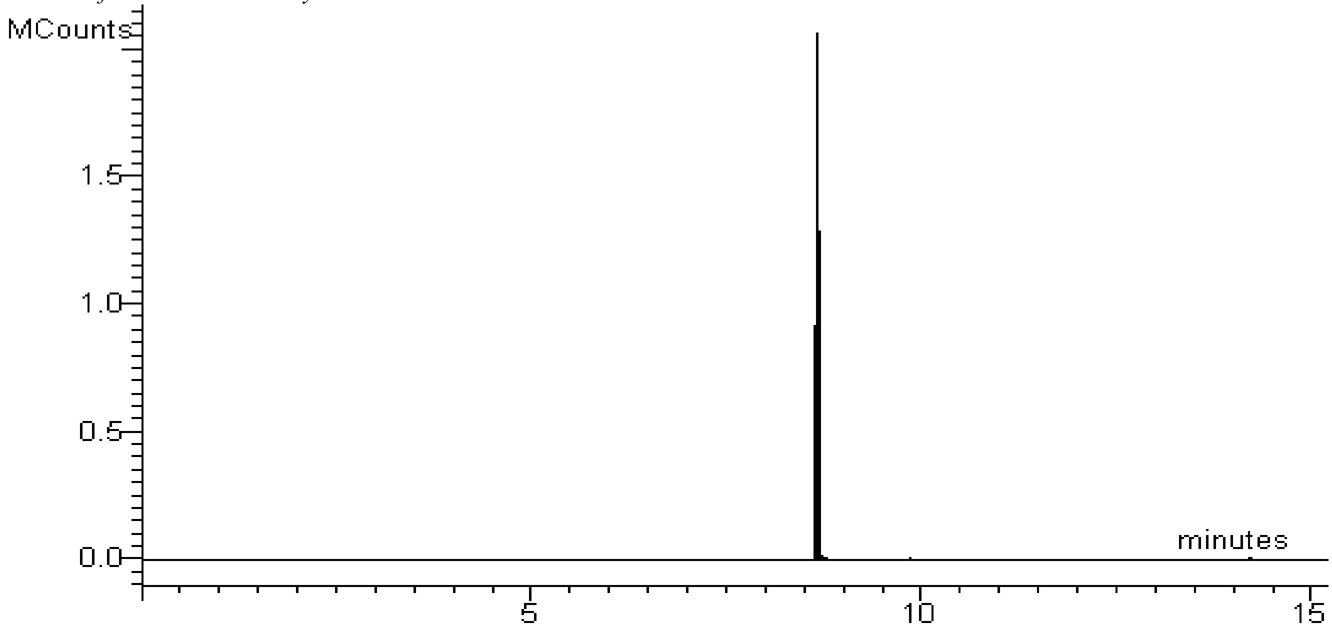

GC trace for distilled propylene carbonate

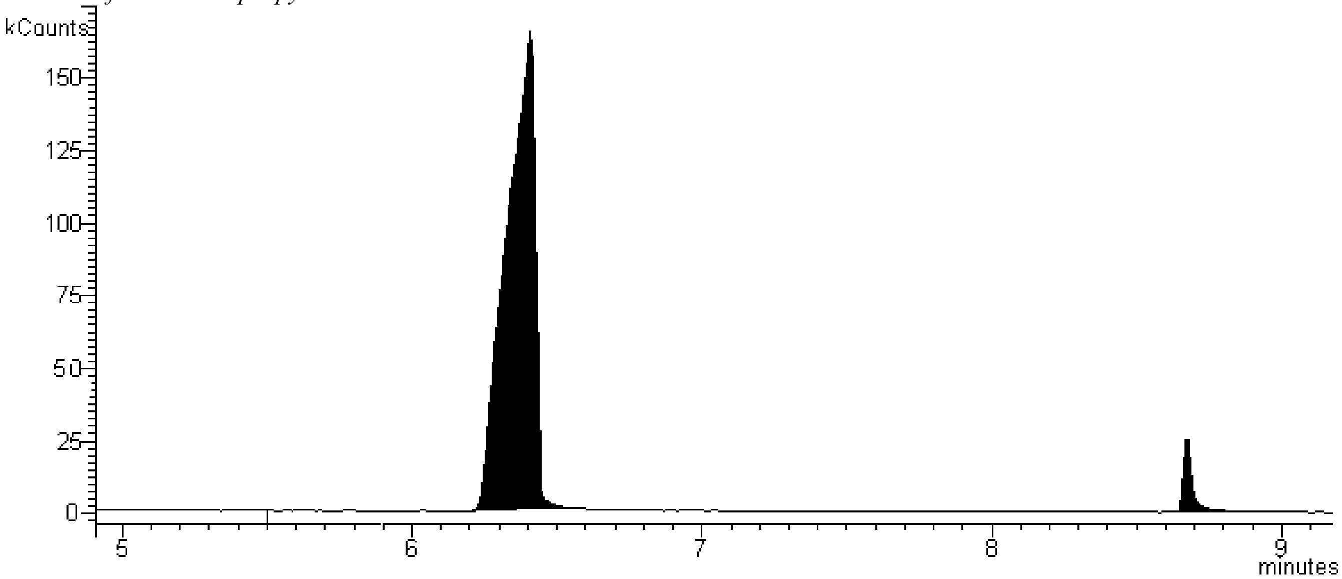

The peak at 6.2-6.5 minutes corresponds to propylene carbonate. The peak at 8.7 minutes corresponds to tributylamine.

EI Mass spectrum of peak at 8.7 minutes (full spectrum)

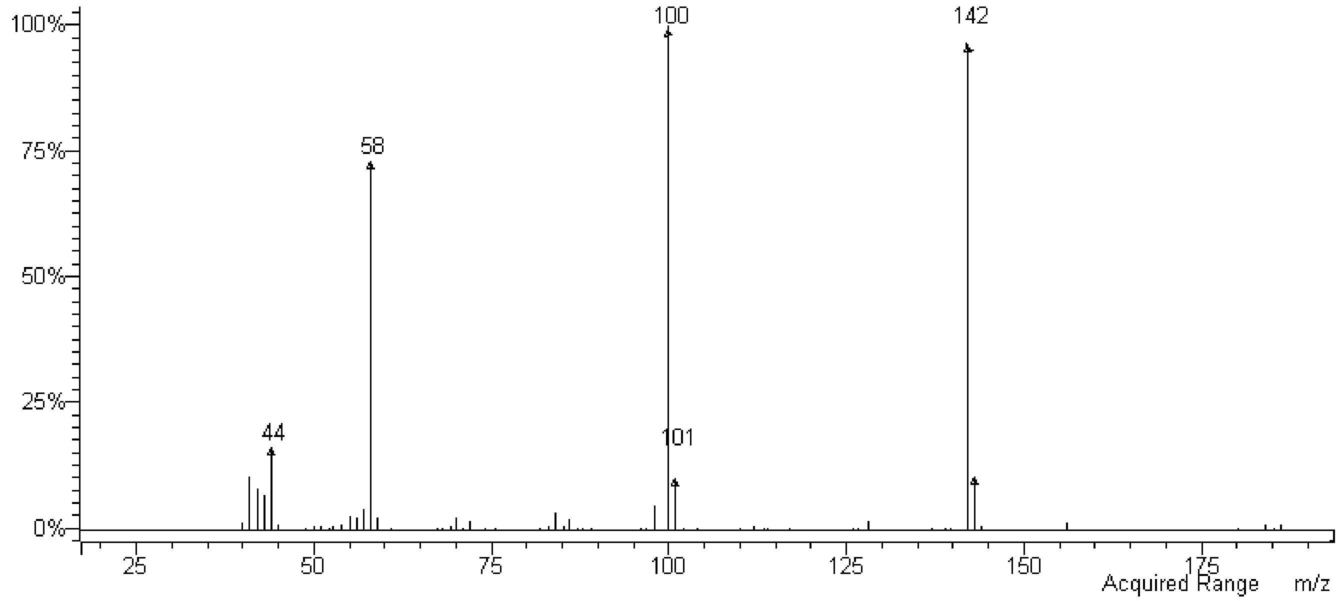

EI Mass spectrum of peak at 8.7 minutes (expansion of m/z 140 to 200)

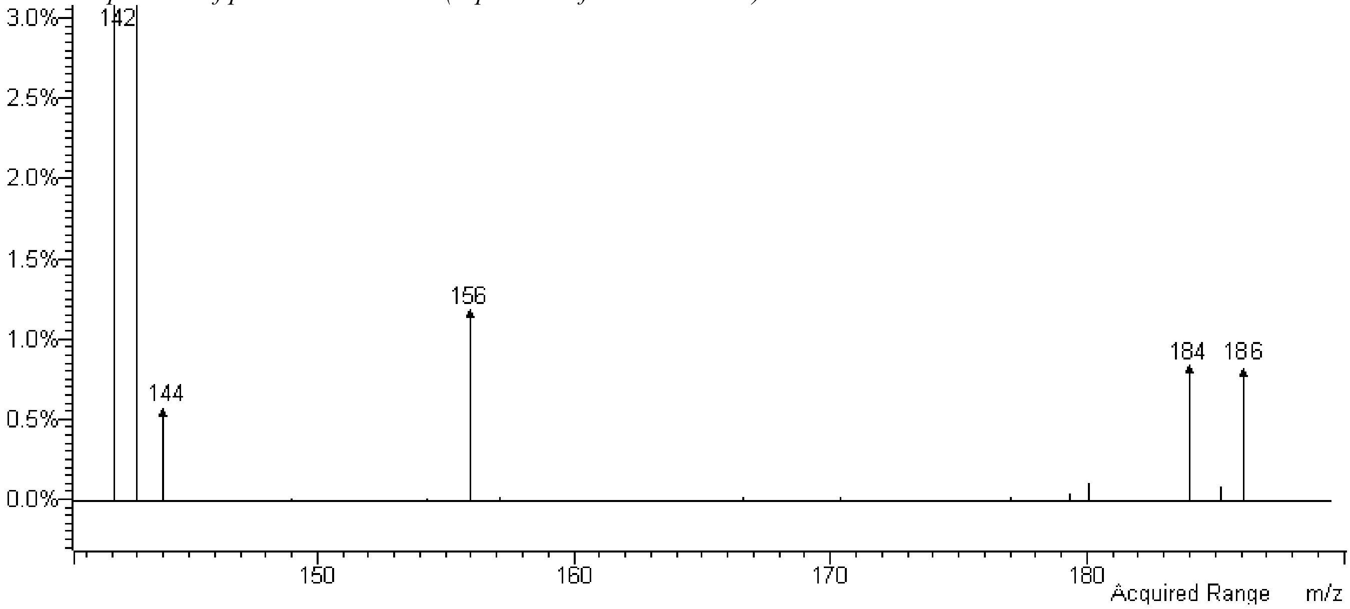

EI Mass spectrum of pure tributylamine

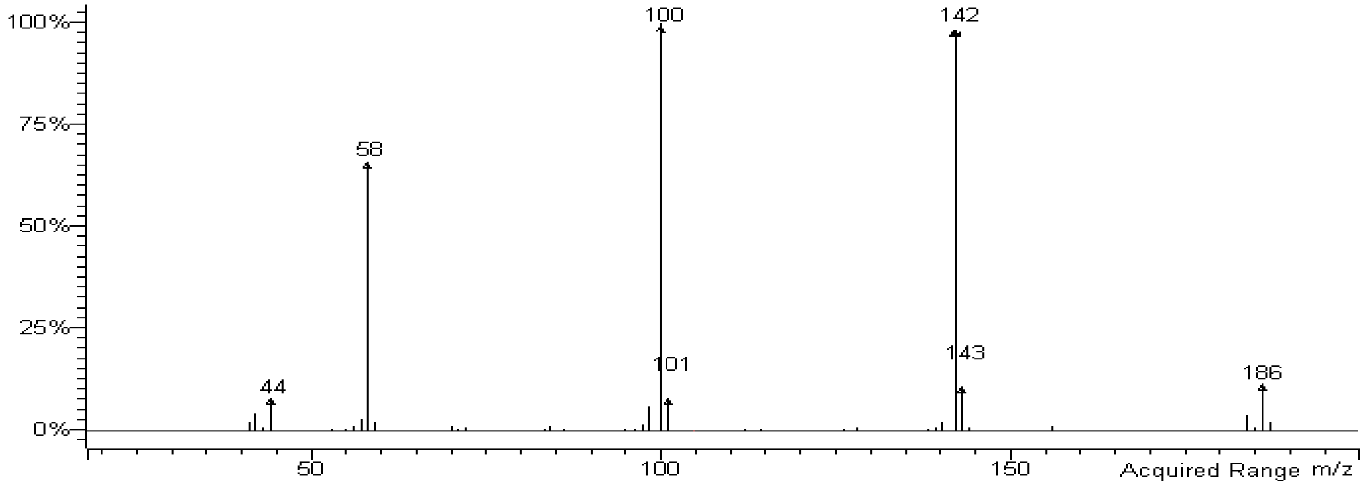

Sequential experiments showing the relationship between product formation and tributylamine presence

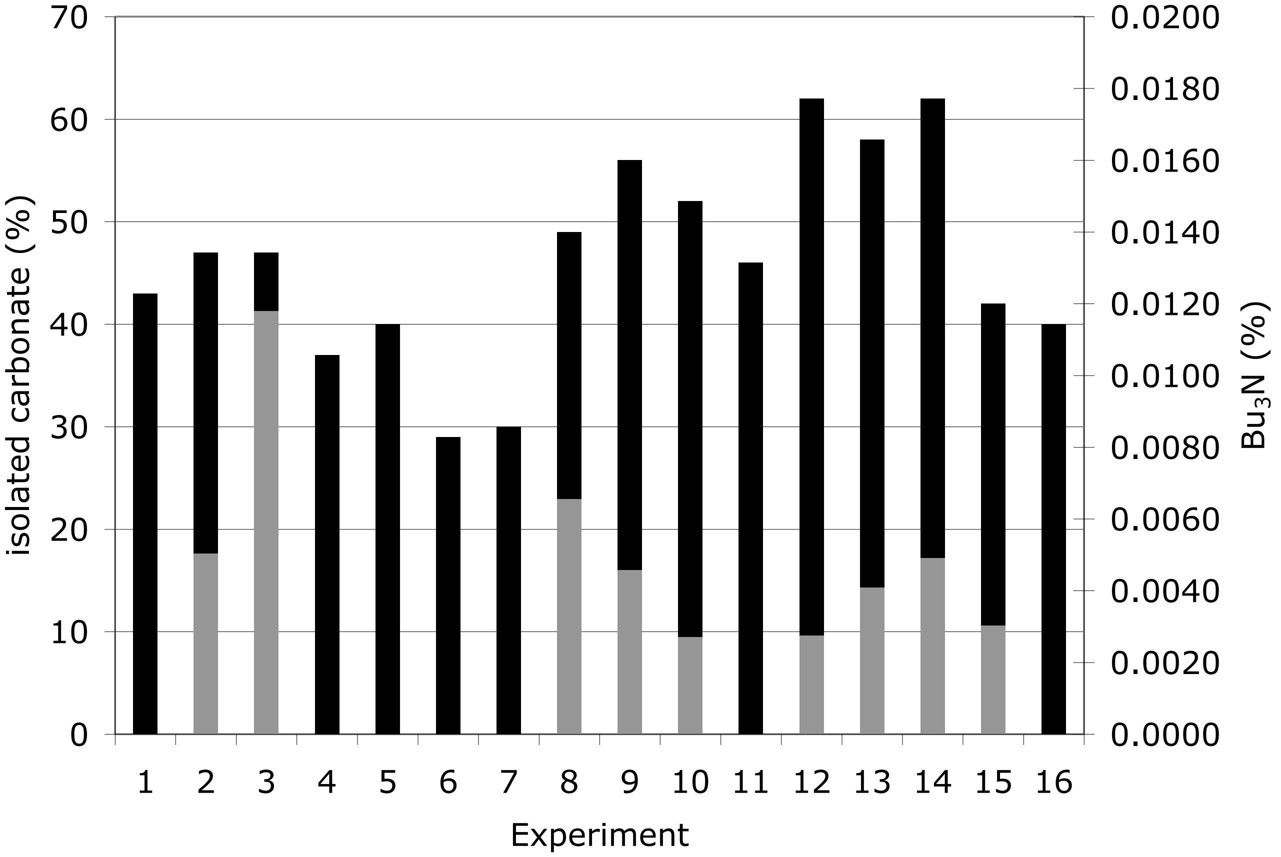

Black column is % yield of propylene carbonate read against the left y-axis. Grey column is % tributylamine detected read against the right y-axis. A second batch of tributylamine (2.5 mol%) was added after the seventh experiment.

Control experiments on the generation of tributylamine with detection by GCMS

Tributylamine can be generated from tetrabutylammonium bromide under high temperature conditions. Since the injector temperature for the GCMS is 250 °C, tetrabutylammonium bromide could form tributylamine during GCMS analysis rather than during a reaction. To investigate this, the following control experiments were carried out:

Tetrabutylammonium bromide (0.005 g, 0.01 mmol) was dissolved in 10 mL of ethyl acetate (10 mL). The mixture was left to stir for 10 minutes, then the solution was analysed by GCMS and tributylamine was detected, showing that if tetrabutylammonium bromide is present in the material injected into the GCMS then tributylamine will be detected.

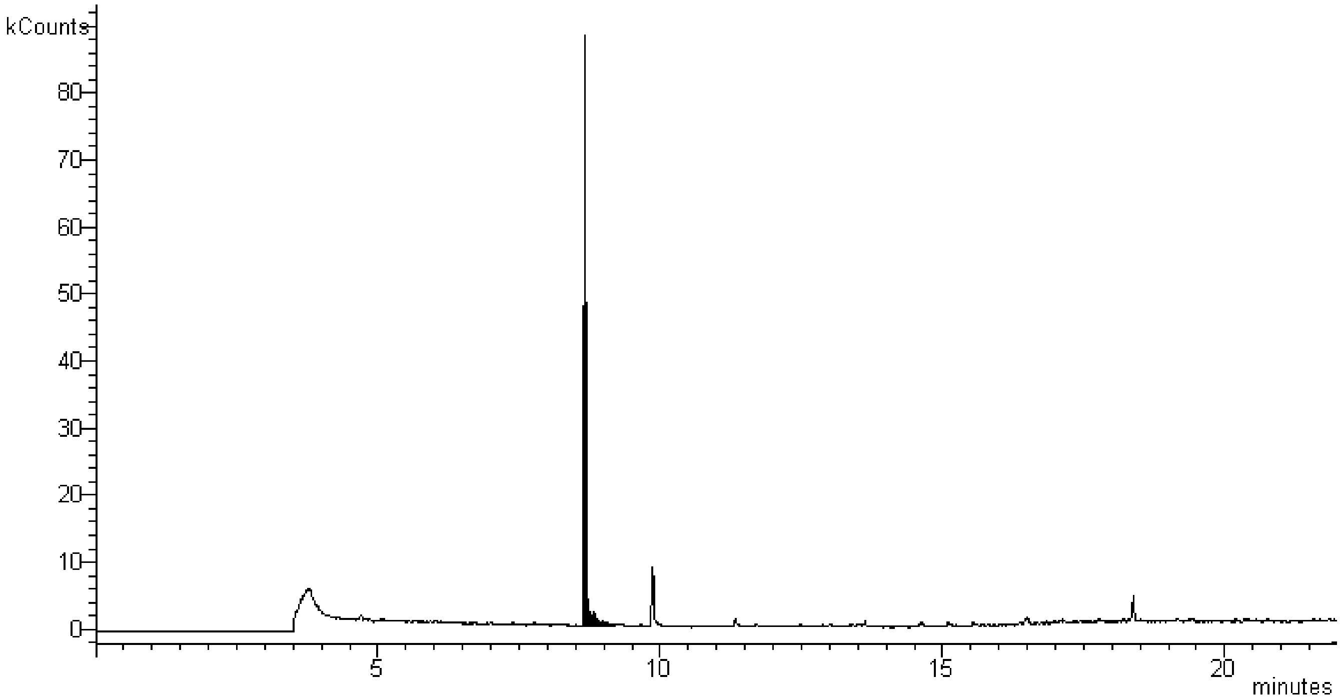

The peak at 8.7 minutes corresponds to tributylamine (authentic GC and m/z data on pages 11 and 12)

Tetrabutylammonium bromide (0.11 g, 0.36 mmol) was dissolved in propylene carbonate (0.48 g, 3.28 mmol) and then stirred until complete dissolution occurred. The mixture was then transferred into a 5 mL flask and purified by micro-distillation (yield 93%, 0.45 g). A sample of the distillate was analysed by GCMS, tributylamine was not detected. Thus tetrabutylammonium bromide does not decompose to tributylamine during the distillation process and there is no splash over of tetrabutylammonium bromide during the distillation. The same result was obtained if complex **1** was also added to the propylene carbonate solution of tetrabutylammonium bromide.

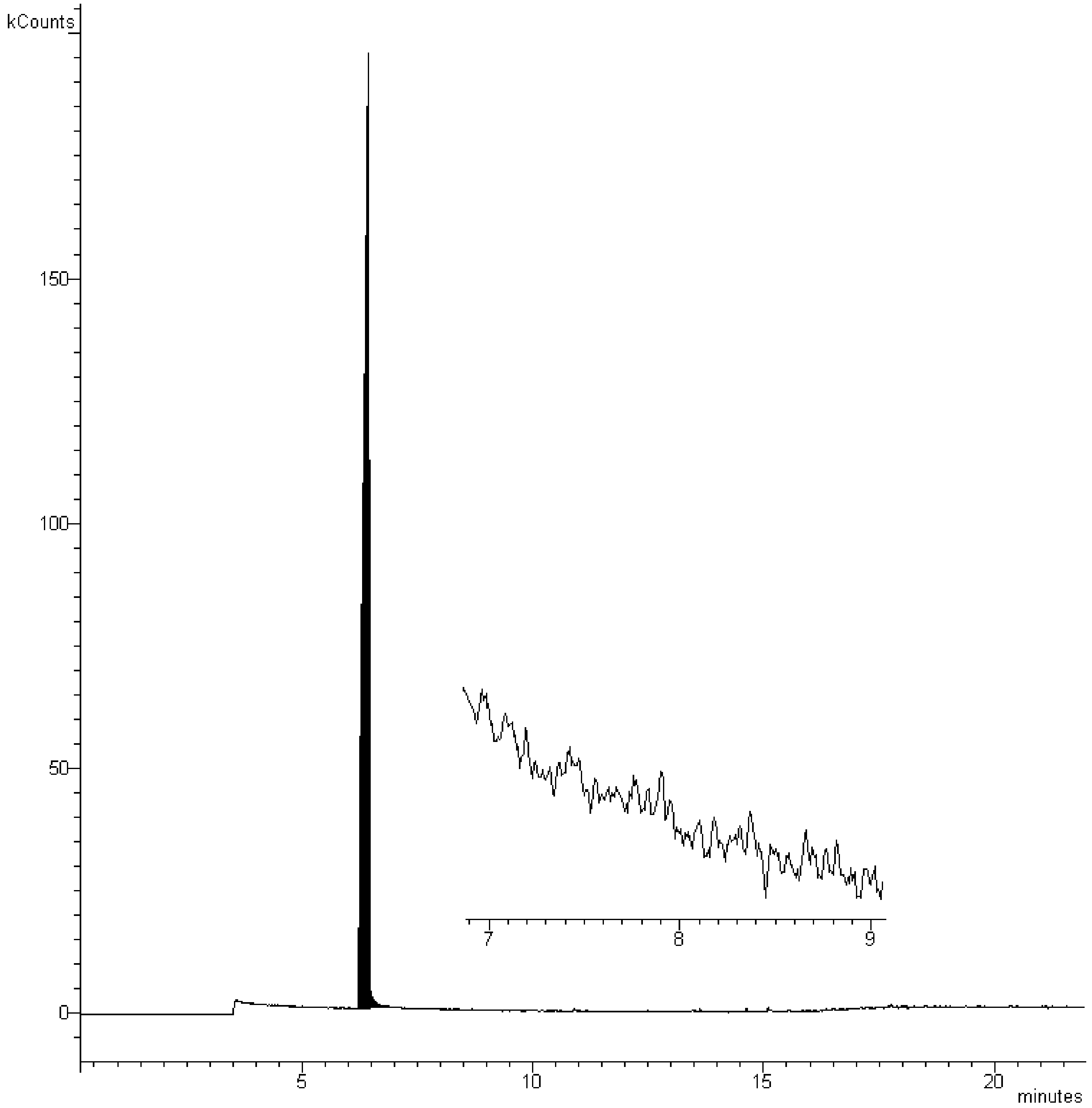

The peak at 6.5 minutes corresponds to propylene carbonate (see data on page 11) and the expansion of 7-9 minutes shows no evidence for tributylamine at 8.7 minutes.

Mass spectra of catalyst 1 before and after its use in 16 consecutive reactions

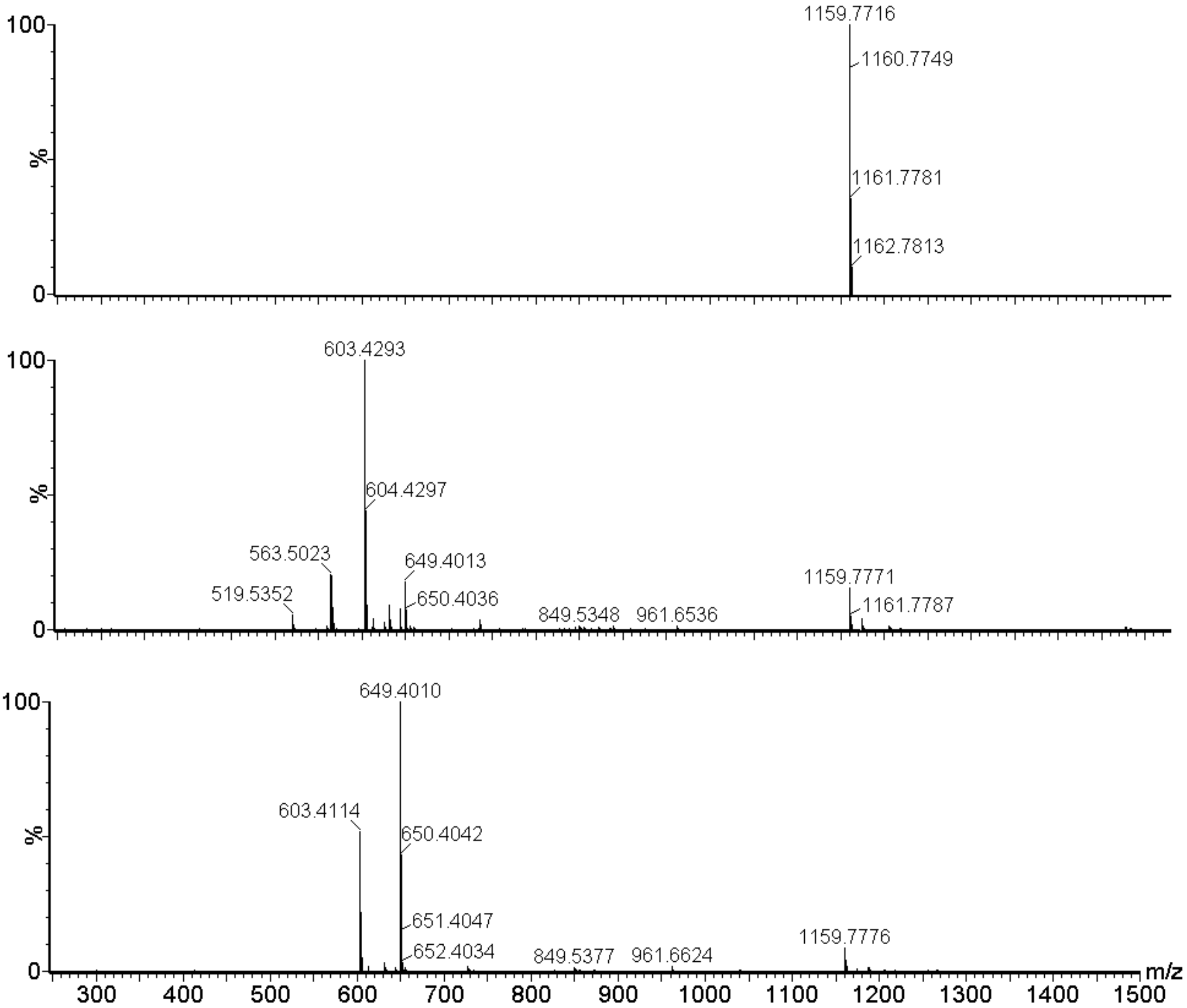

Top: theoretical data for **1** (MH^+).
Bottom: freshly prepared sample of catalyst **1**.
Middle: crude sample of catalyst **1** isolated after being used in 16 consecutive reactions to convert propylene oxide into propylene carbonate.

The peaks at m/z 603 and 649 correspond to $[(salen)AlOMe+H]^+$ and $[(salen)AlOMe+H+Na_2]^+$ respectively formed by reaction of complex **1** with methanol within the LCMS system.
